# Supplementary material for: Real-time 31P NMR reveals different gradient strengths in polyphosphoester copolymers as potential MRI-traceable nanomaterials
Source: Commun Chem. 2023 Sep 1;6:182. doi: 10.1038/s42004-023-00954-x (PMC10474120; doi:10.1038/s42004-023-00954-x)
Supplement: Supplementary file 2 — Supplementary Information [file 42004_2023_954_MOESM2_ESM.pdf]

Supplementary Information

Real-time ^{31}P NMR reveals different gradient strengths in polyphosphoester copolymers as potential MRI-traceable nanomaterials

*Timo Rheinberger,[1] Ulrich Flögel,[2] Olga Koshkina,[1] Frederik R. Wurm[1]**

1) Sustainable Polymer Chemistry (SPC), Department of Molecules and Materials, MESA+ Institute for Nanotechnology, Faculty of Science and Technology, University of Twente, P.O. Box 217, 7500 AE Enschede, Netherlands; f.r.wurm@utwente.nl
2) Department of Molecular Cardiology, Experimental Cardiovascular Imaging, Heinrich-Heine-University, Düsseldorf, Germany.

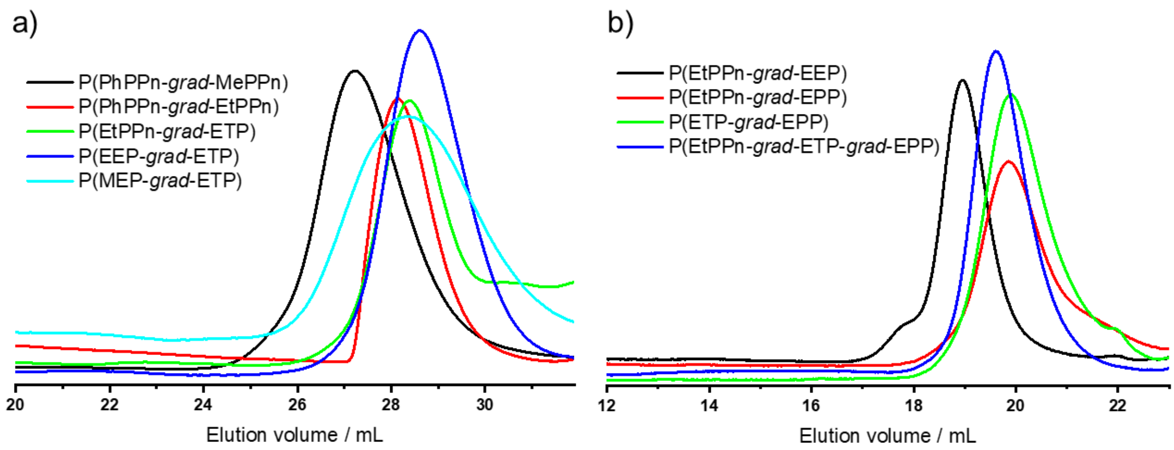

Figure S1: GPC elugrams of the prepared copolymers measured in DMF (0.1 M LiCl, at 50 °C), a) carried out on a three column set, b) measured on a two column set.

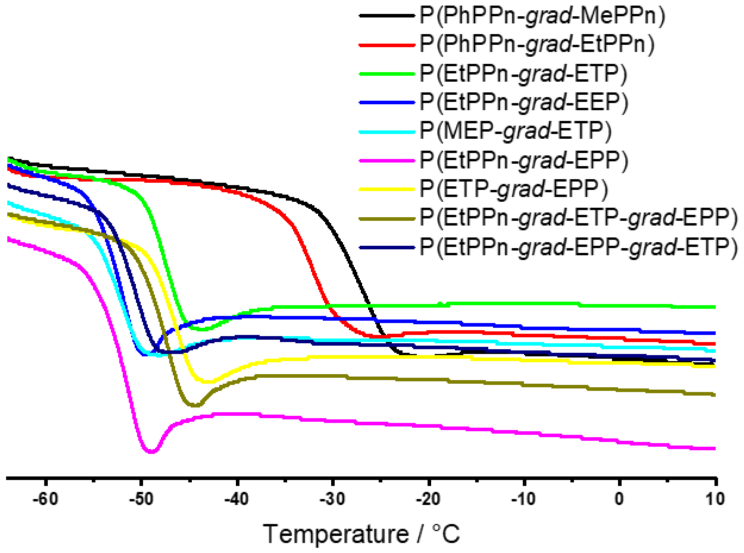

Figure S2: Differential scanning calorimetry (DSC) measurements of different PPE copolymers (shown is the second heating curve (10 K/min), exo up).

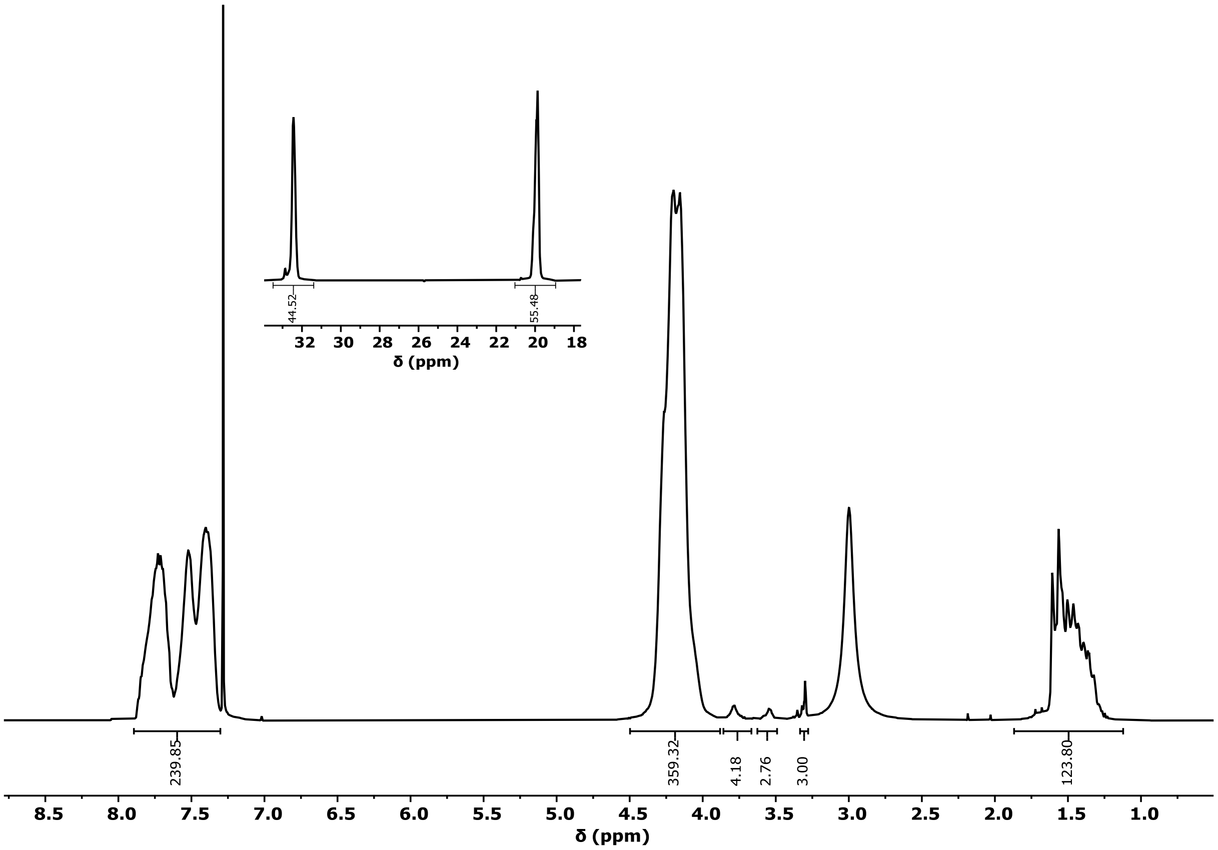

Figure S3: Representative ^{1}H NMR (400 MHz, 298 K, $CDCl_3$) and $^{31}P\{H\}$ NMR (162 MHz, 298 K, $CDCl_3$) (inset) spectra of polymer P1 P(PhPPn-*grad*-EtPPn) polymerised in DCM with DBU inside of the NMR tube after purification.

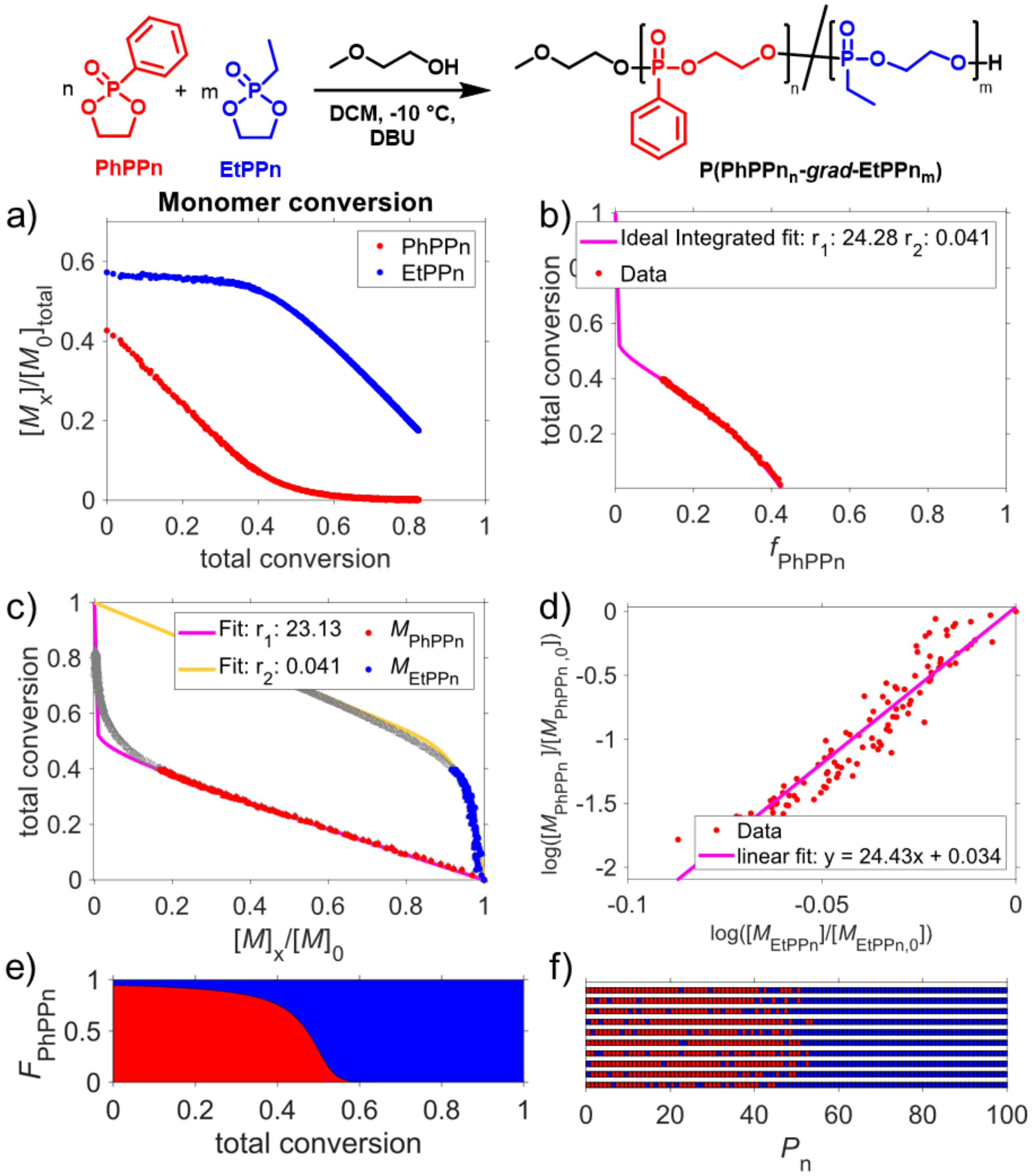

Figure S4: Kinetics data and calculation of reactivity ratios: Data from the copolymerisation of PhPPn and EtPPn (entry **P2**, Table 1) to P(PhPPn-*grad*-EtPPn), a) monomer concentration as function of total conversion of PhPPn and EtPPn; for fitting the data up to 40 % of total conversion were used for all three models, b) Ideal integrated fit of real-time NMR data of monomers, c) Jaacks fit of real-time NMR data of monomers, d) BSL fit of real-time NMR data of monomers, e) Visualization of the copolymer compositions by a plot of the average monomer fraction composition against the total conversion, f) 10 discrete polymer chains calculated via Monte Carlo simulation using the determined reactivity ratios.

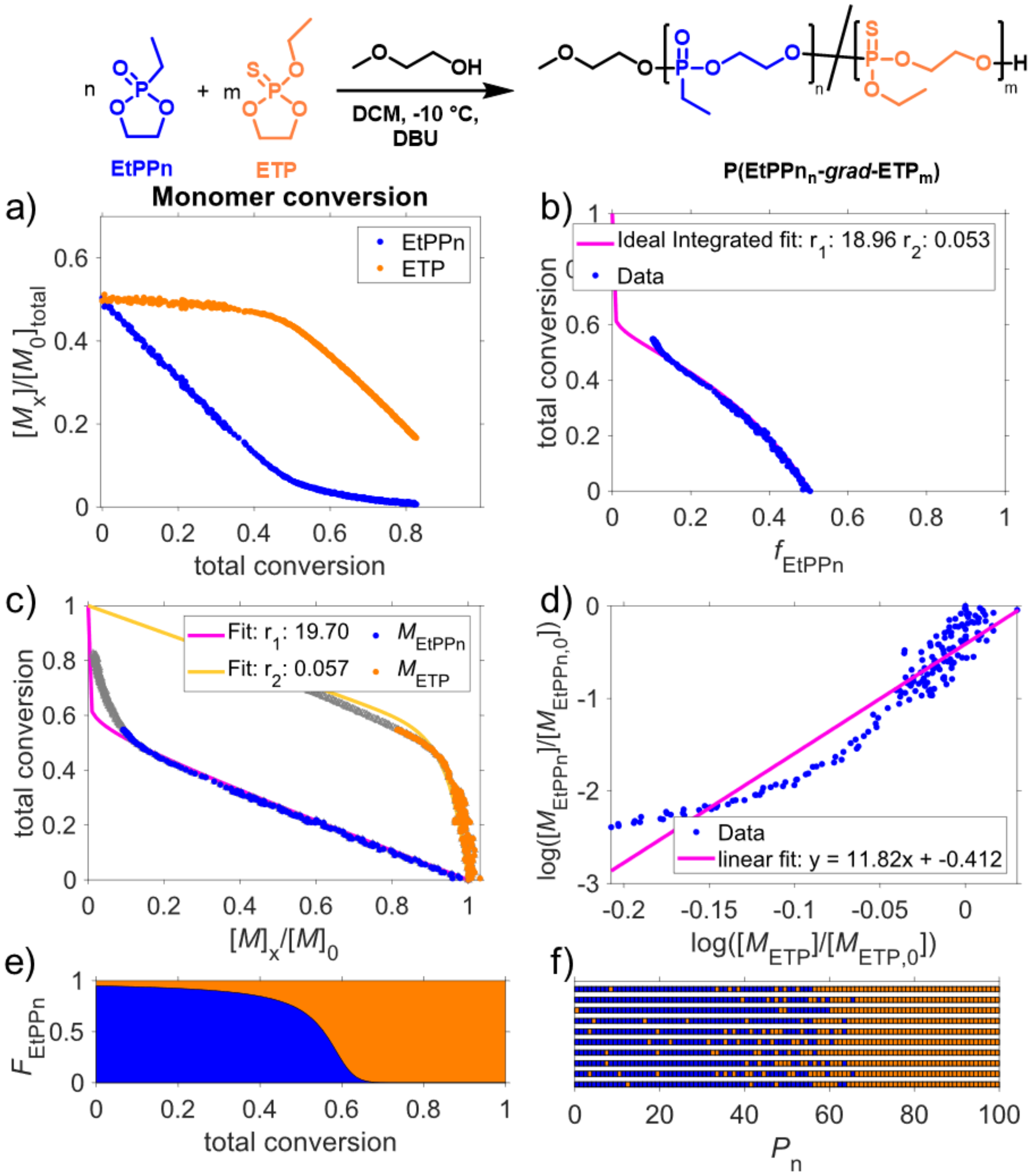

Figure S5: Kinetics data and calculation of reactivity ratios: Data from the copolymerisation of EtPPn and ETP (entry **P3**, Table 1) to P(EtPPn-*grad*-ETP), a) monomer concentration as function of total conversion of EtPPn and ETP; for fitting the data up to 55 % of total conversion were used for all three models, b) Ideal integrated fit of real-time NMR data of monomers, c) Jaacks fit of real-time NMR data of monomers, d) BSL fit of real-time NMR data of monomers, e) Visualization of the copolymer compositions by a plot of the average monomer fraction composition against the total conversion, f) 10 discrete polymer chains calculated via Monte Carlo simulation using the determined reactivity ratios.

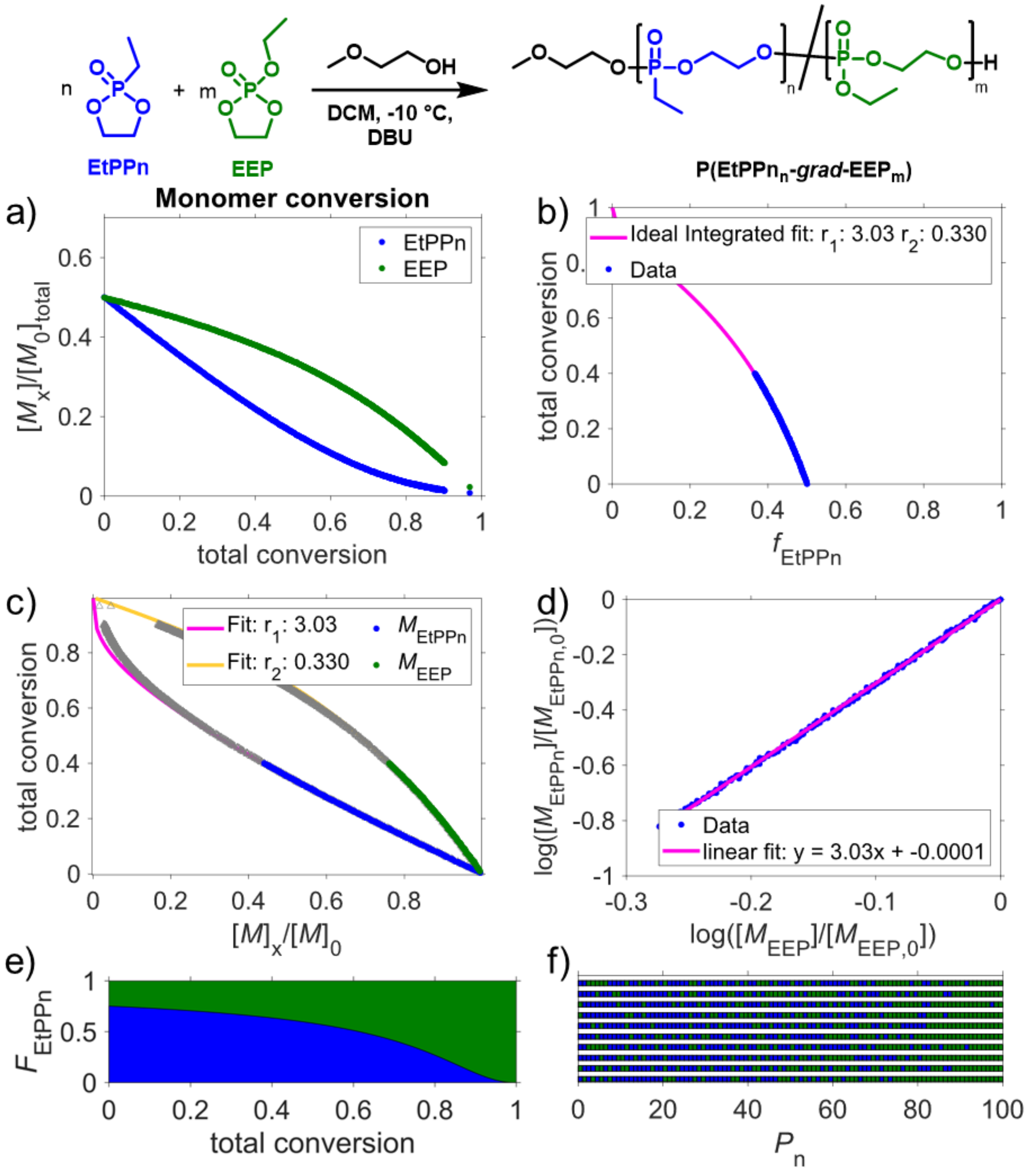

Figure S6: Kinetics data and calculation of reactivity ratios: Data from the copolymerisation of EtPPn and EEP (entry **P4**, Table 1) to P(EtPPn-*grad*-EEP), a) monomer concentration as function of total conversion of EtPPn and EEP; for fitting the data up to 40 % of total conversion were used for all three models, b) Ideal integrated fit of real-time NMR data of monomers, c) Jaacks fit of real-time NMR data of monomers, d) BSL fit of real-time NMR data of monomers, e) Visualization of the copolymer compositions by a plot of the average monomer fraction composition against the total conversion, f) 10 discrete polymer chains calculated via Monte Carlo simulation using the determined reactivity ratios.

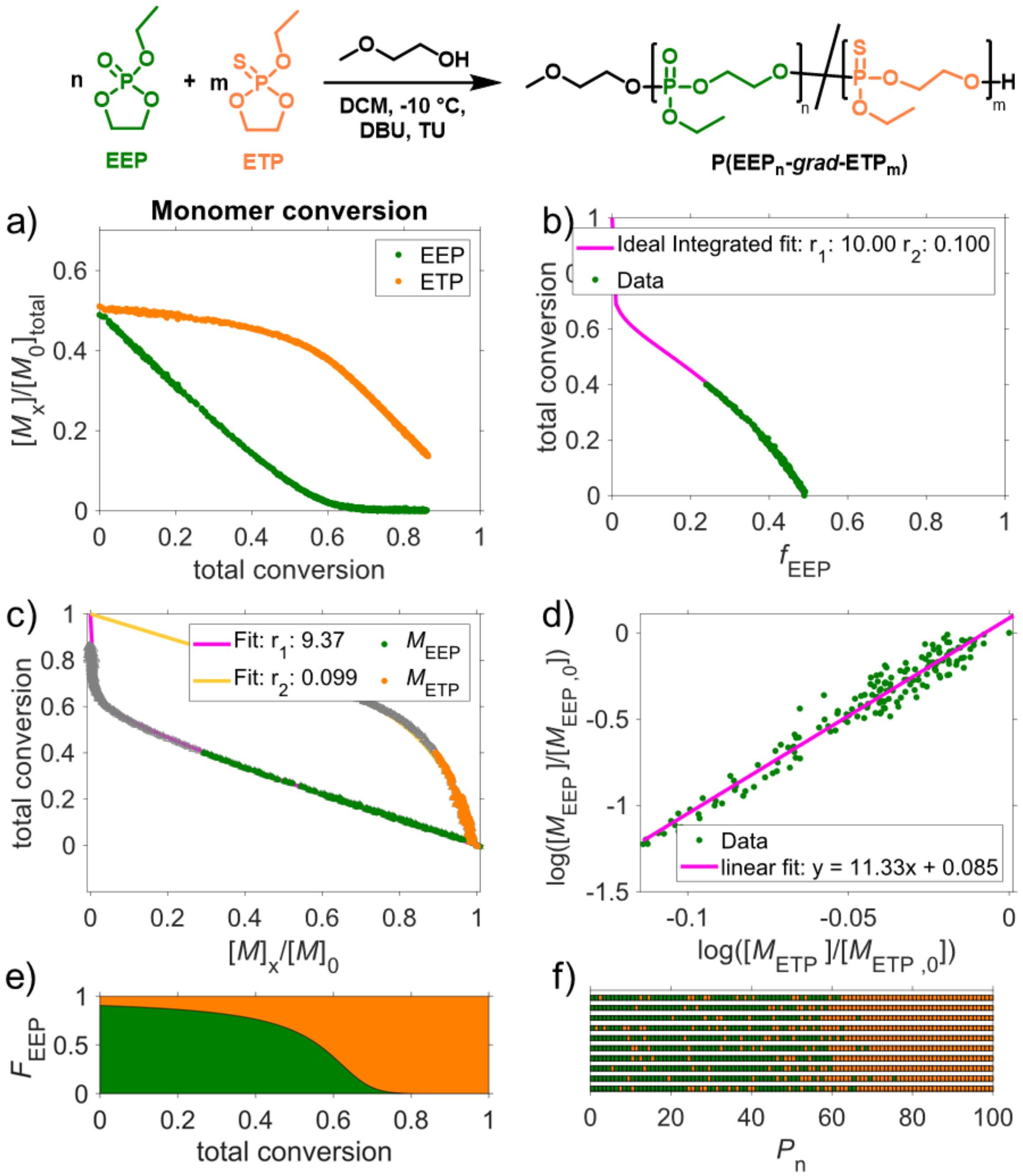

Figure S7: Kinetics data and calculation of reactivity ratios: Data from the copolymerisation of EEP and ETP (entry **P5**, Table 1) to P(EEP-*grad*-ETP), a) monomer concentration as function of total conversion of EEP and ETP; for fitting the data up to 40 % of total conversion were used for all three models, b) Ideal integrated fit of real-time NMR data of monomers, c) Jaacks fit of real-time NMR data of monomers, d) BSL fit of real-time NMR data of monomers, e) Visualization of the copolymer compositions by a plot of the average monomer fraction composition against the total conversion, f) 10 discrete polymer chains calculated via Monte Carlo simulation using the determined reactivity ratios.

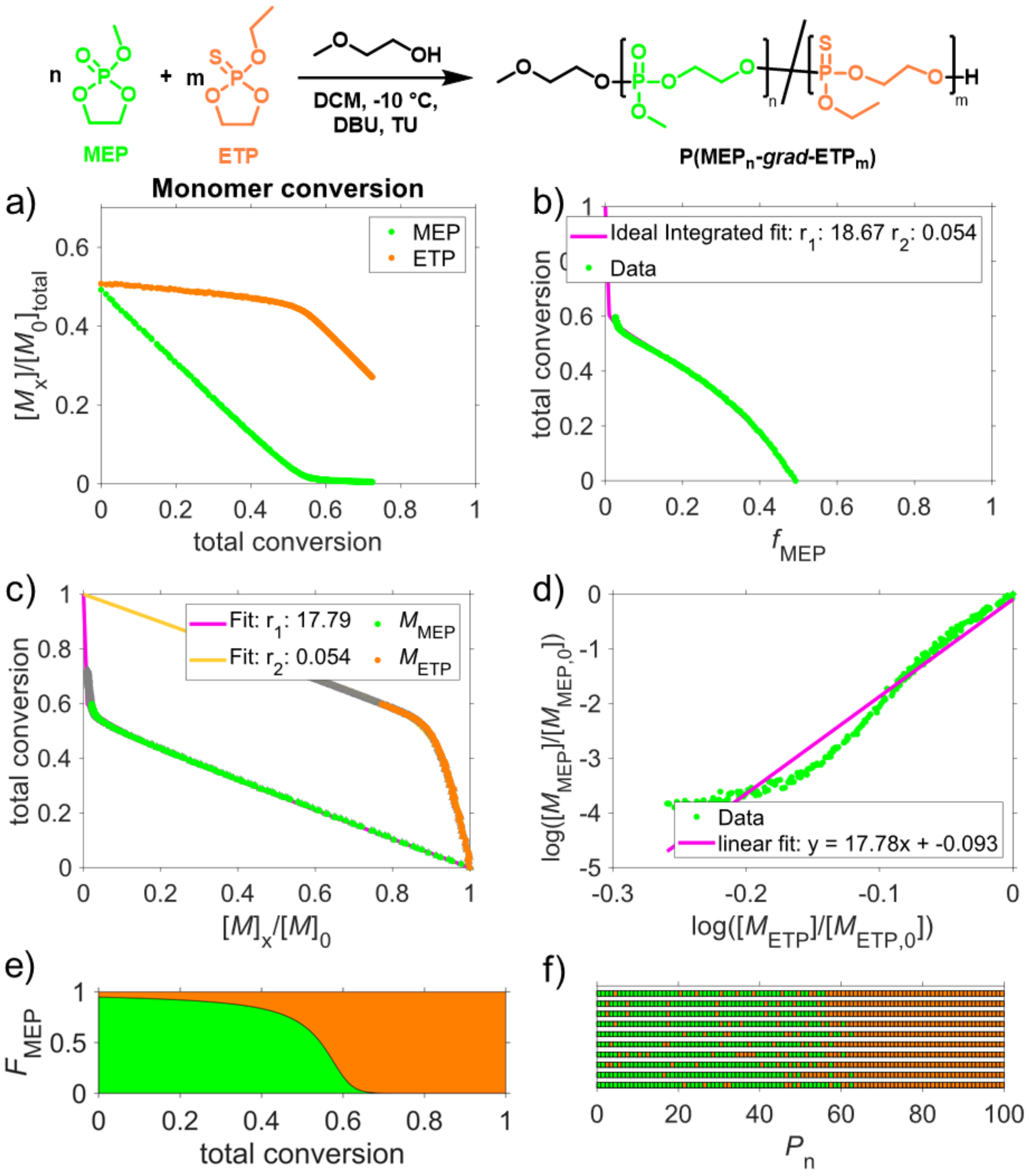

Figure S8: Kinetics data and calculation of reactivity ratios: Data from the copolymerisation of MEP and ETP (entry **P6**, Table 1) to P(MEP-*grad*-ETP), a) monomer concentration as function of total conversion of MEP and ETP; for fitting the data up to 60 % of total conversion were used for all three models, b) Ideal integrated fit of real-time NMR data of monomers, c) Jaacks fit of real-time NMR data of monomers, d) BSL fit of real-time NMR data of monomers, e) Visualization of the copolymer compositions by a plot of the average monomer fraction composition against the total conversion, f) 10 discrete polymer chains calculated via Monte Carlo simulation using the determined reactivity ratios.

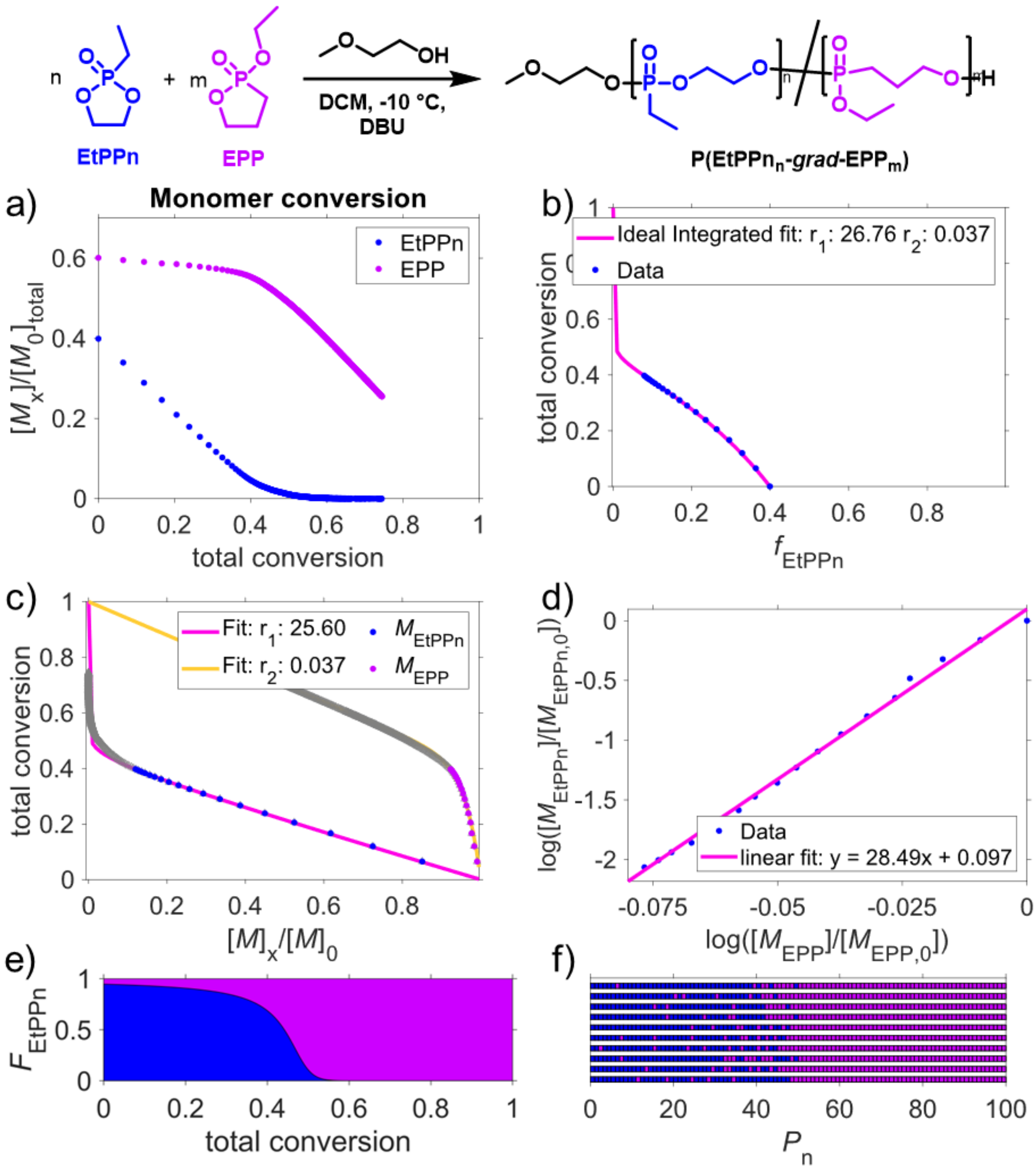

Figure S9: Kinetics data and calculation of reactivity ratios: Data from the copolymerisation of EtPPn and EPP (entry **P7**, Table 1) to P(EtPPn-*grad*-EPP), a) monomer concentration as function of total conversion of EtPPn and EPP; for fitting the data up to 40 % of total conversion were used for all three models, b) Ideal integrated fit of real-time NMR data of monomers, c) Jaacks fit of real-time NMR data of monomers, d) BSL fit of real-time NMR data of monomers, e) Visualization of the copolymer compositions by a plot of the average monomer fraction composition against the total conversion, f) 10 discrete polymer chains calculated via Monte Carlo simulation using the determined reactivity ratios.

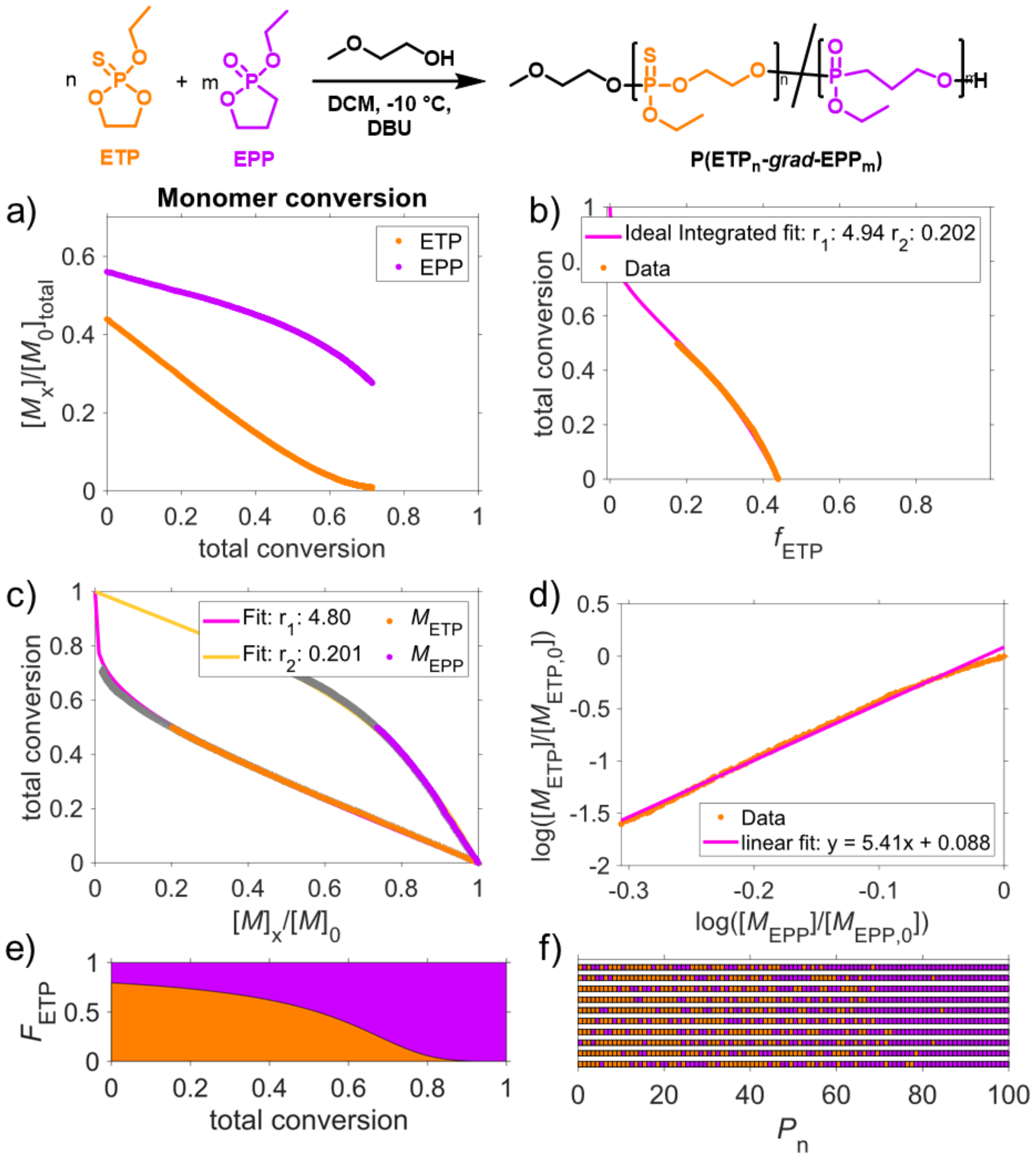

Figure S10: Kinetics data and calculation of reactivity ratios: Data from the copolymerisation of ETP and EPP (entry **P8**, Table 1) to P(ETP-*grad*-EPP), a) monomer concentration as function of total conversion of ETP and EPP; for fitting the data up to 50 % of total conversion were used for all three models, b) Ideal integrated fit of real-time NMR data of monomers, c) Jaacks fit of real-time NMR data of monomers, d) BSL fit of real-time NMR data of monomers, e) Visualization of the copolymer compositions by a plot of the average monomer fraction composition against the total conversion, f) 10 discrete polymer chains calculated via Monte Carlo simulation using the determined reactivity ratios.

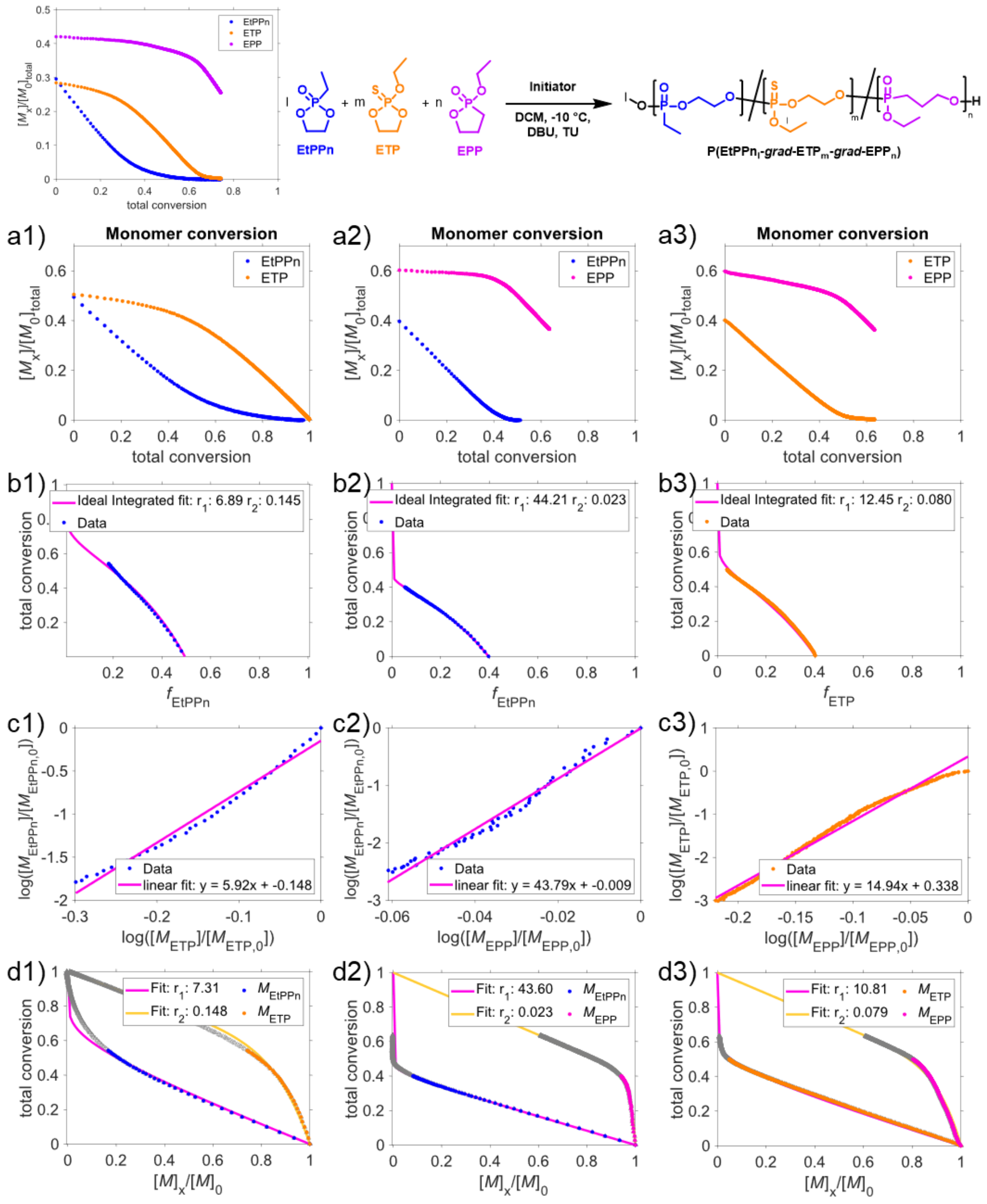

Figure S11: Kinetics data and calculation of reactivity ratios with three different monomers: Data from the copolymerisation of EtPPn, ETP and EPP (entry P9, Table 1) to P(EtPPn-*grad*-ETP-*grad*-EPP), a) monomer concentration as function of "total conversion" of a1) EtPPn and ETP, a2) EtPPn and EPP, a3) ETP and EPP; for the fitting data up to 1) 55 %, 2) 40 % and 3) 50 % of total conversion were used for all three models, b1-3) Ideal integrated fit of real-time NMR data of monomers, c1-3) Jaacks fit of real-time NMR data of monomers, d1-3) BSL fit of real-time NMR data of monomers.

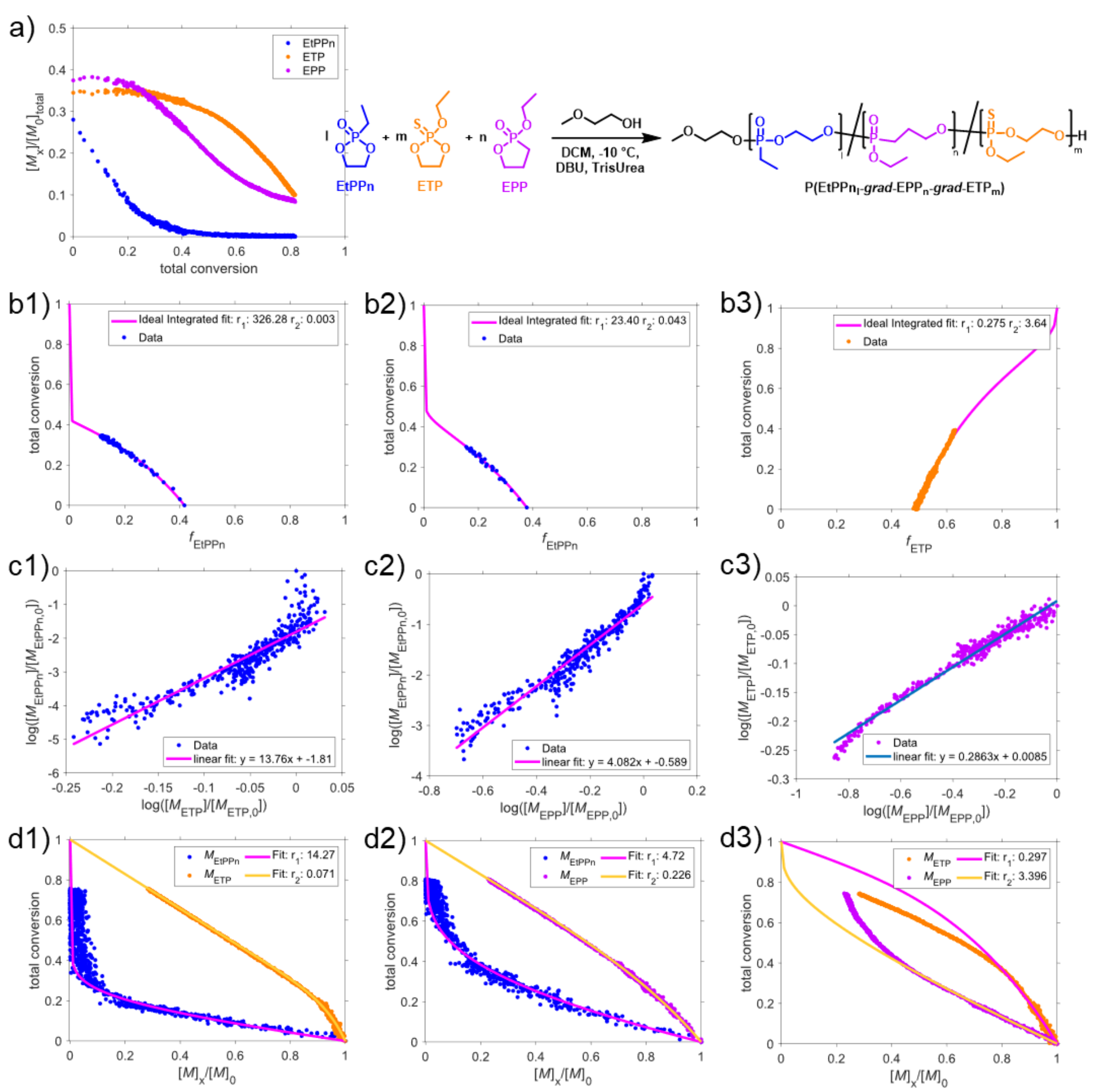

Figure S12: Kinetics data and calculation of reactivity ratios with three different monomers: Data from the copolymerisation of EtPPn, ETP and EPP (entry P10, Table 1) to P(EtPPn-*grad*-ETP-*grad*-EPP), a) monomer concentration as function of total conversion of EtPPn, ETP and EPP; for the fitting data up to 60 % were used for all three models an individual fit data range optimization was done to determine the best fit, b1-3) Ideal integrated fit of real-time NMR data of monomers, c1-3) Jaacks fit of real-time NMR data of monomers, d1-3) BSL fit of real-time NMR data of monomers.

Table S1: Summary of the polymer properties in aqueous solution and interfacial properties cyclohexane/water.

	Polymers	D_h [a]	error	Inter facial tension LY [b] / mN·m^{-1}					relaxation times [c]					
#	P(X-*grad*-Y-*grad*-Z)	/ nm	/ nm	0.1 g·L^{-1}	1 g·L^{-1}	10 g·L^{-1}	average	error	T_1 (X)	T_2 (X)	T_1 (Y)	T_2 (Y)	T_1 (Z)	T_2 (Z)
P1	P(PhPPn-*grad*-MePPn)	22	1	14.52	14.05	13.61	14.1	0.5	0.9	0.077	1.97	0.41	-	-
P2	P(PhPPn-*grad*-EtPPn)	34	2	13.84	13.79	13.26	13.6	0.3	0.96	0.15	1.96	0.81	-	-
P3	P(EtPPn-*grad*-ETP)	321	74	12.68	11.46	10.08	11	1	1.68	0.72	0.55	0.114	-	-
P4	P(EtPPn-*grad*-EEP)	-*	-	21.49	21.20	18.84	20	1	2.4	0.79	2.2	0.74	-	-
P5	P(EEP-*grad*-ETP)	265	1	11.77	10.99	9.84	10	1	1.13	0.089	0.65	0.011	-	-
P6	P(MEP-*grad*-ETP)	5	1	n.d.	n.d.	n.d.	-	-	1.63	0.574	0.66	0.044	-	-
P7	P(EtPPn-*grad*-EPP)	-*	-	20.91	20.53	14.92	18	3	2.52	1.32	1.96	1.74	-	-
P8	P(ETP-*grad*-EPP)	176	12	14.66	10.23	8.46	11	3	0.71	0.0065	1.25	0.03	-	-
P9	P(EtPPn-*grad*-ETP-*grad*-EPP)	130	19	9.19	7.95	7.13	8	1	1.46	0.28	0.58	0.026	1.04	0.10
P10	P(EtPPn-*grad*-EPP-*grad*-ETP)	38	6	n.d.	n.d.	n.d.	-	-	2.15	0.72	1.43	0.31	0.58	0.03

a) measured by DLS at 90° angle, b) determined by sinning drop measurement c) relaxation times were measured in H_2O:D_2O/ 9:1 at 9.4 T (172 MHz for ^{31}P); n.d. not determined; * the double hydrophilic polymers are water-soluble and show to low scattering intensity.
