# Supplementary material for: Real-time 31P NMR reveals different gradient strengths in polyphosphoester copolymers as potential MRI-traceable nanomaterials
Source: Commun Chem. 2023 Sep 1;6:182. doi: 10.1038/s42004-023-00954-x (PMC10474120; doi:10.1038/s42004-023-00954-x)
Supplement: Supplementary file 3 — Description of Additional Supplementary Files [file 42004_2023_954_MOESM3_ESM.pdf]

Description of Additional Supplementary Files

File name: Supplementary Data 1

Description: 1H and 13C NMR Spectra of the final polymers
