# Supplementary material for: Real-time 31P NMR reveals different gradient strengths in polyphosphoester copolymers as potential MRI-traceable nanomaterials
Source: Commun Chem. 2023 Sep 1;6:182. doi: 10.1038/s42004-023-00954-x (PMC10474120; doi:10.1038/s42004-023-00954-x)
Supplement: Supplementary file 4 — Supplementary Data 1 [file 42004_2023_954_MOESM4_ESM.pdf]

^{1}H and ^{13}C NMR Spectra of the final polymers

P1 P(PhPPn-*grad*-MePPn)

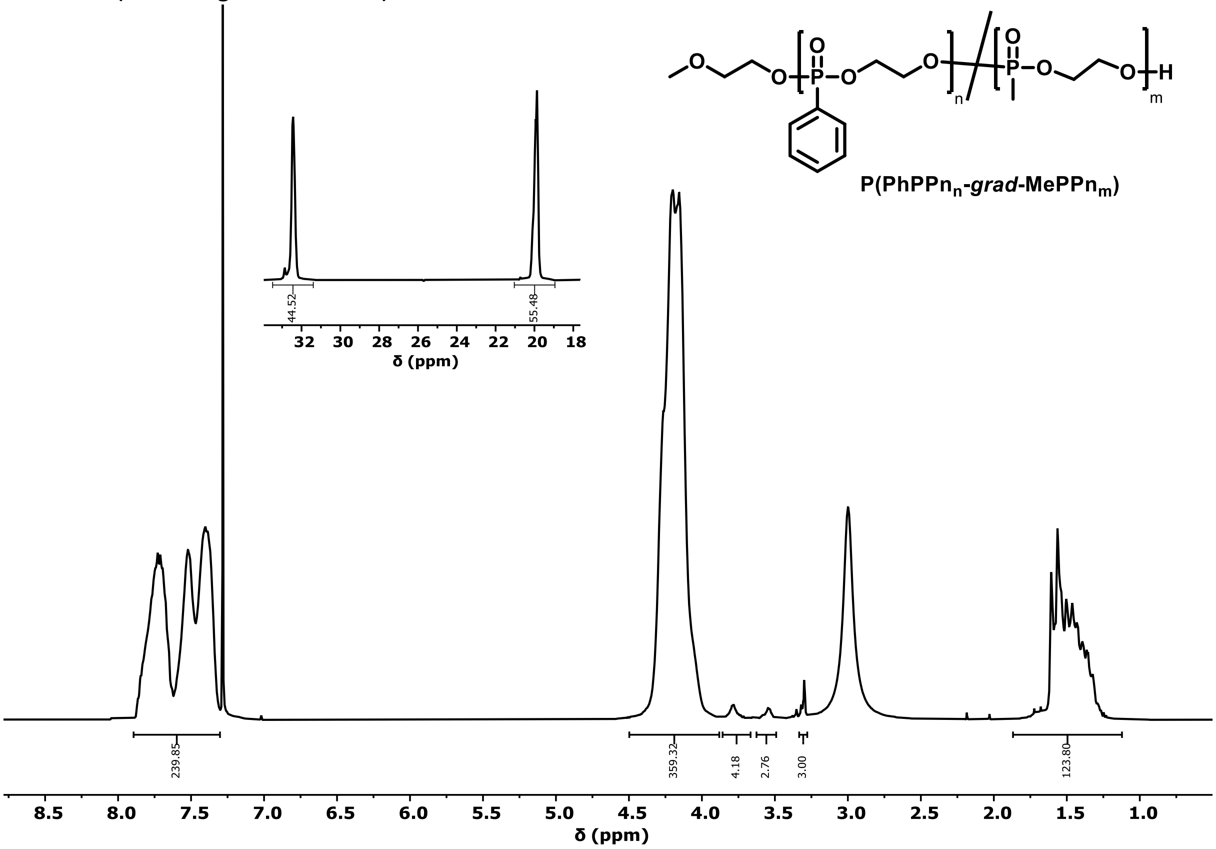

^{1}H NMR (400 MHz, 298 K, $CDCl_3$) and $^{31}P\{H\}$ NMR (162 MHz, 298 K, $CDCl_3$)

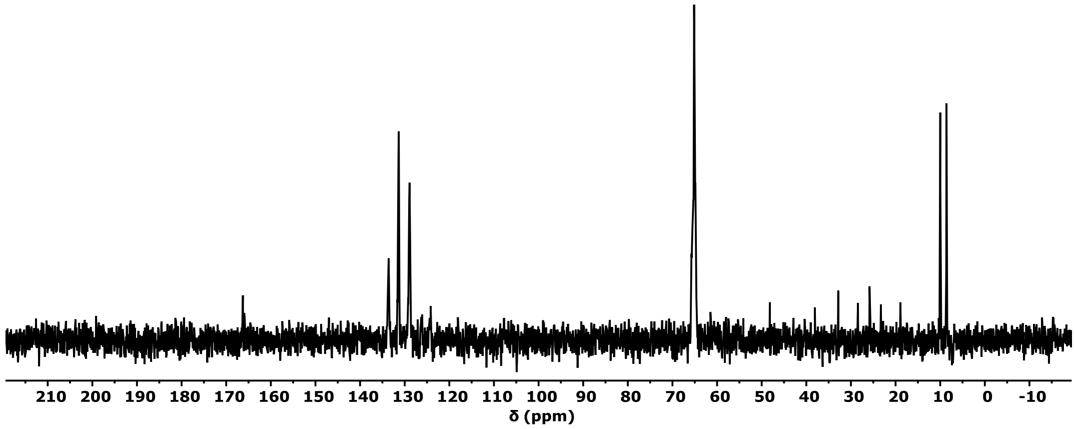

$^{13}C\{H\}$ NMR (101 MHz, 298 K, H_2O/D_2O 9:1)

P2 P(PhPPn-*grad*-EtPPn)

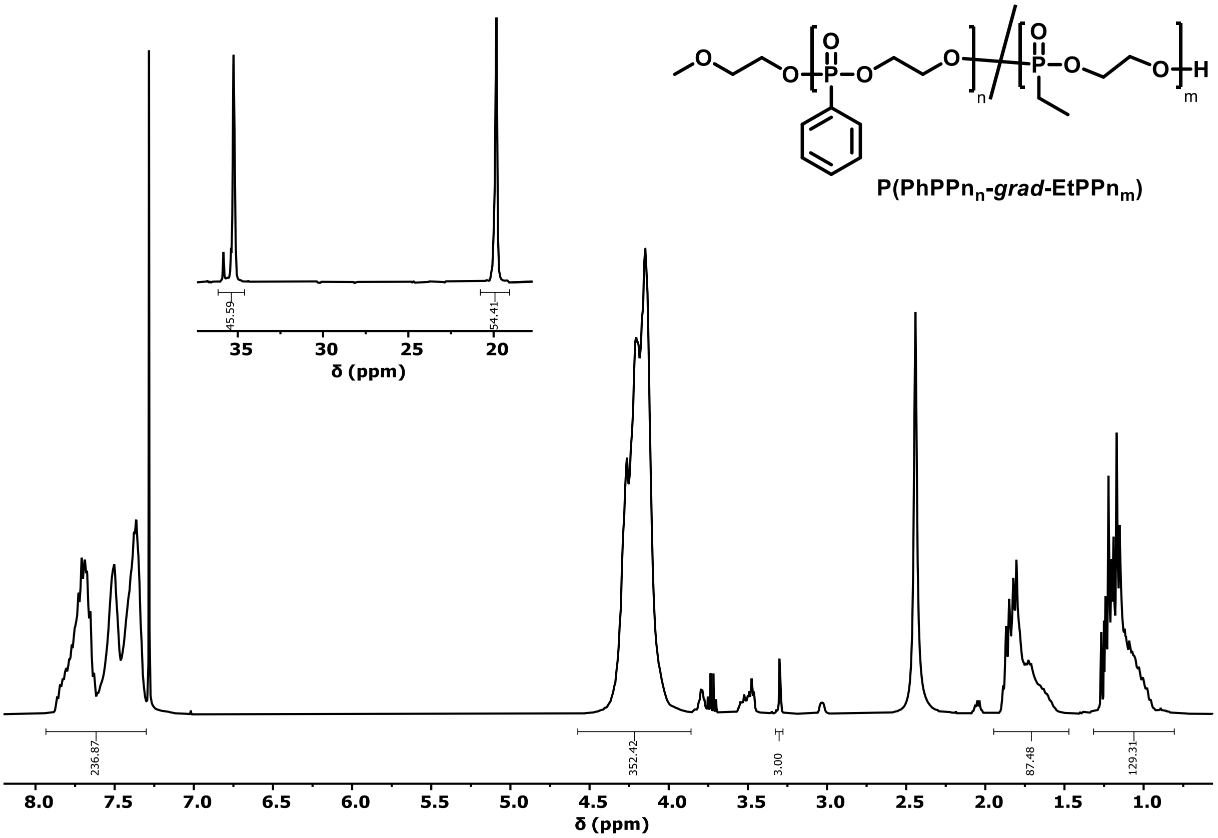

^{1}H NMR (400 MHz, 298 K, $CDCl_3$) and ^{31}P{H} NMR (162 MHz, 298 K, $CDCl_3$)

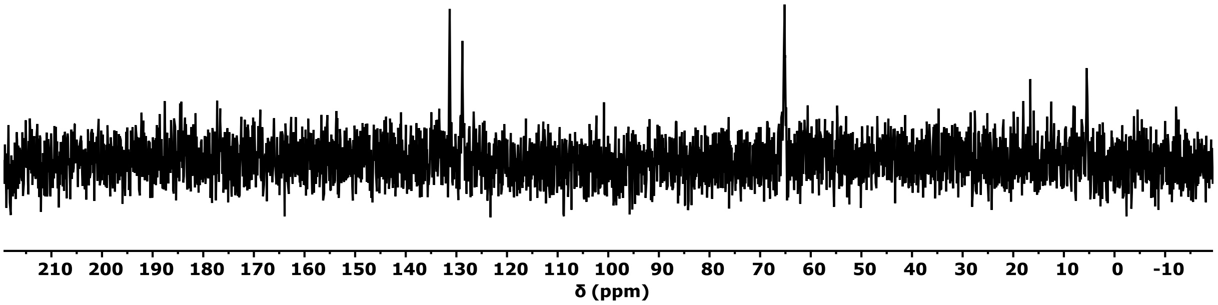

^{13}C{H} NMR (101 MHz, 298 K, H_2O/D_2O 9:1)

P3 P(EtPPn-*grad*-ETP)

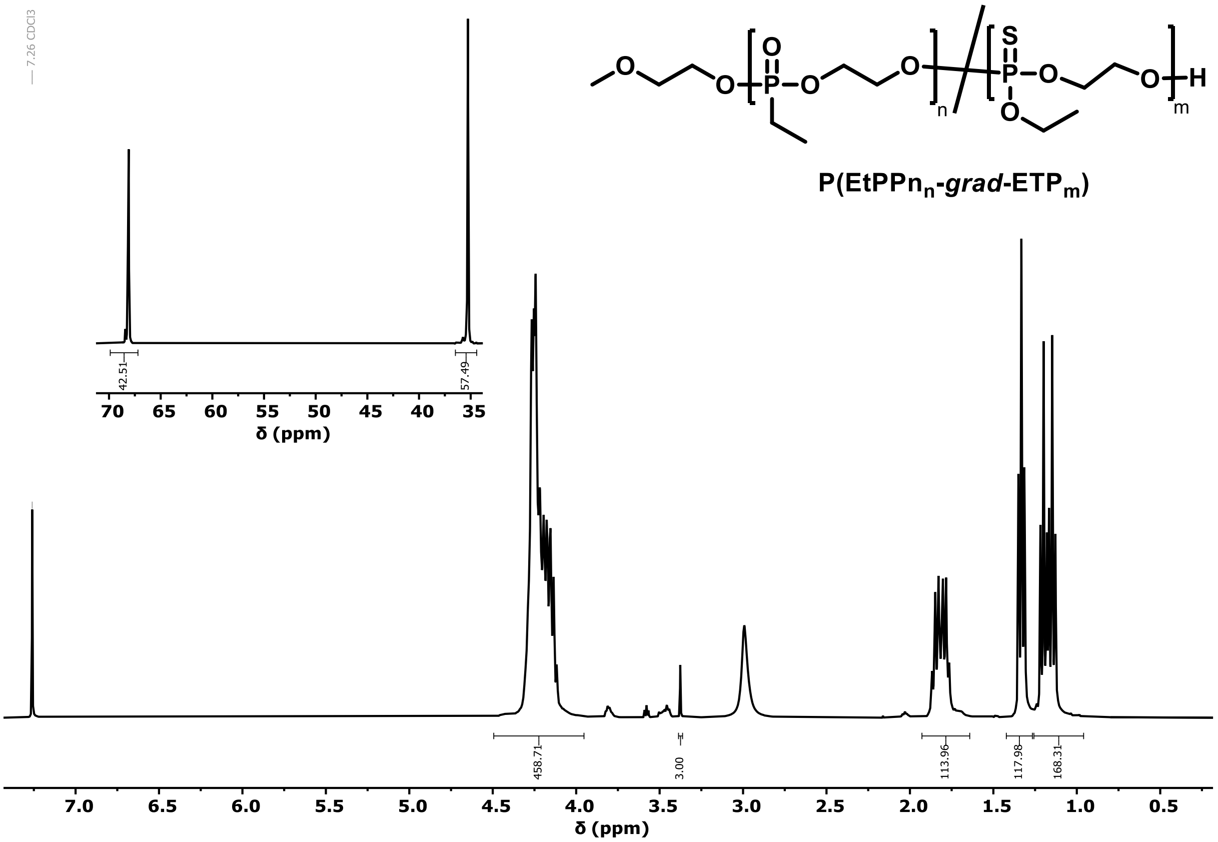

^{1}H NMR (400 MHz, 298 K, $CDCl_3$) and ^{31}P{H} NMR (162 MHz, 298 K, $CDCl_3$)

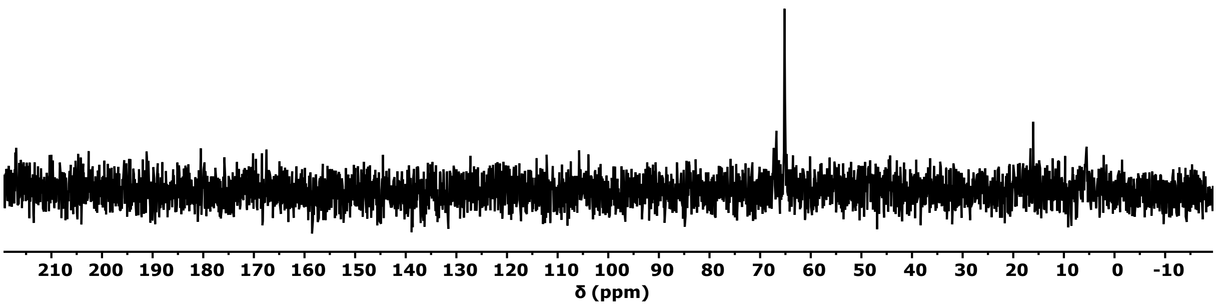

^{13}C{H} NMR (101 MHz, 298 K, H_2O/D_2O 9:1)

P4 P(EtPPn-*grad*-EEP)

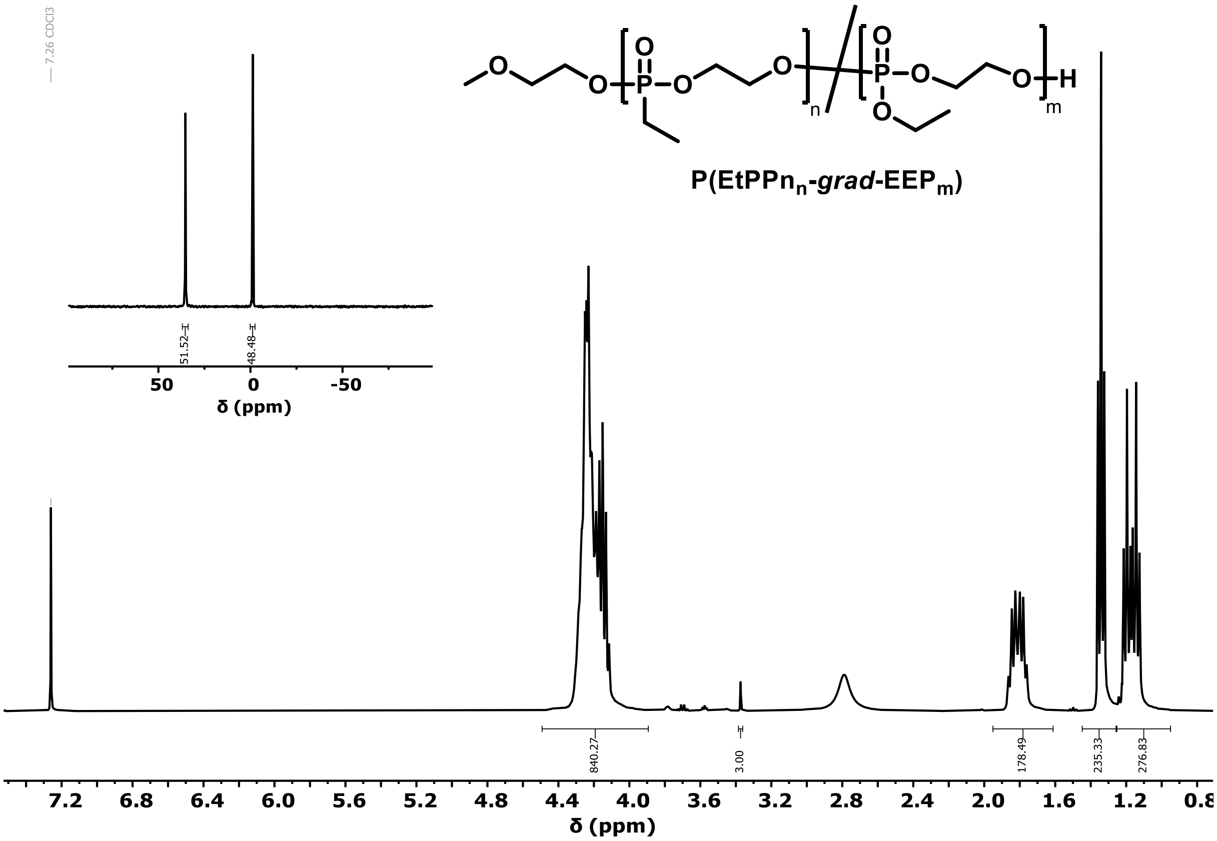

^{1}H NMR (400 MHz, 298 K, $CDCl_3$) and $^{31}P\{H\}$ NMR (162 MHz, 298 K, $CDCl_3$)

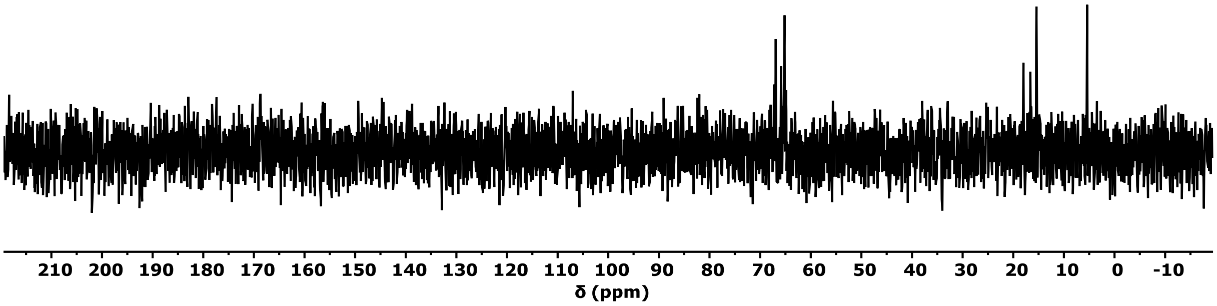

$^{13}C\{H\}$ NMR (101 MHz, 298 K, H_2O/D_2O 9:1)

P5 P(EEP-*grad*-ETP)

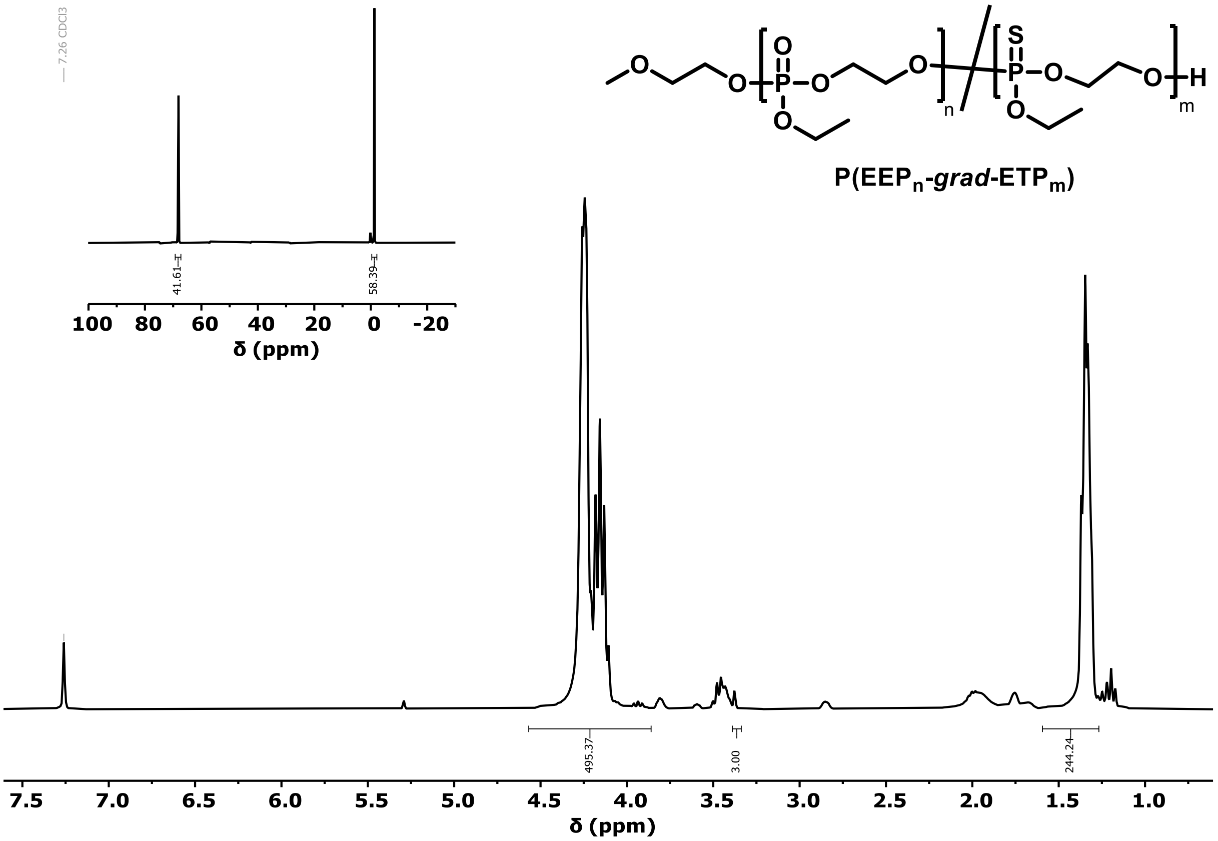

^{1}H NMR (400 MHz, 298 K, $CDCl_3$) and ^{31}P{H} NMR (162 MHz, 298 K, $CDCl_3$)

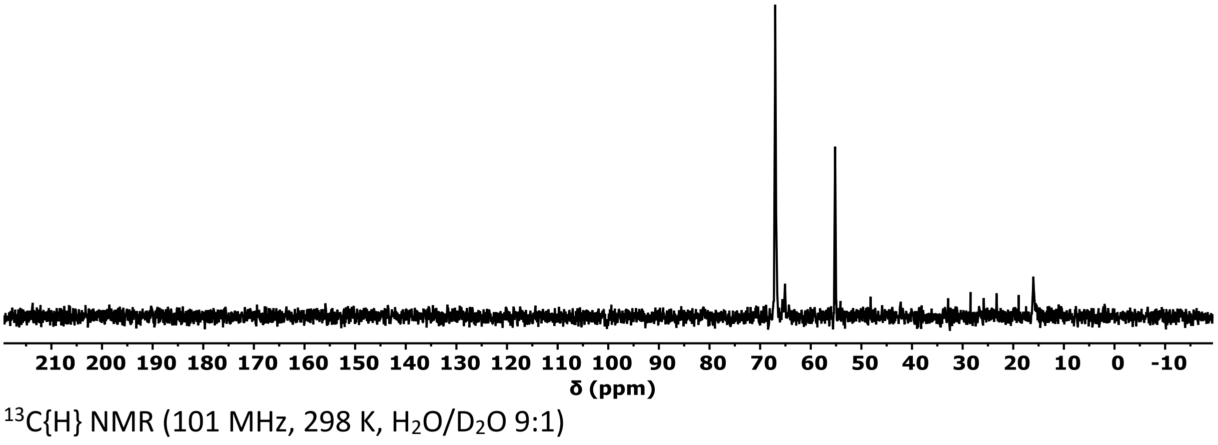

^{13}C{H} NMR (101 MHz, 298 K, H_2O/D_2O 9:1)

P6 P(MEP-*grad*-ETP)

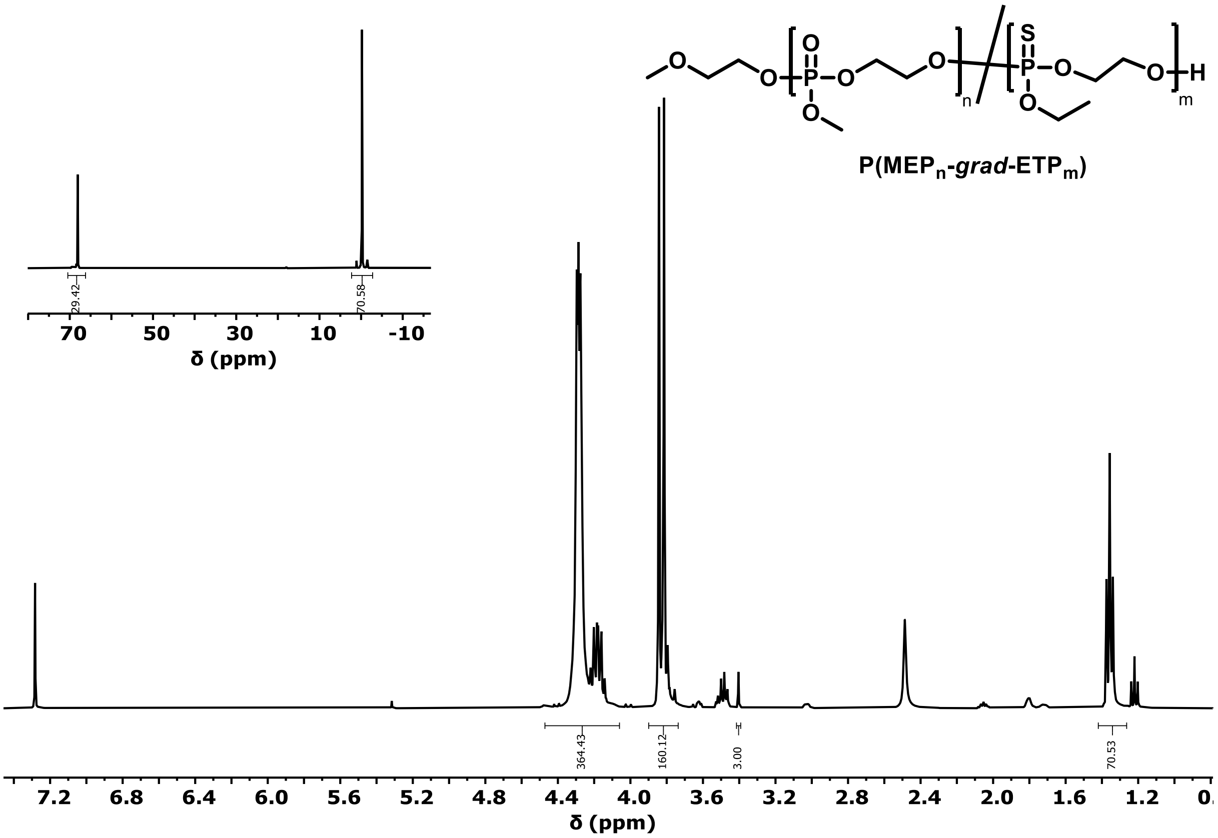

^{1}H NMR (400 MHz, 298 K, $CDCl_3$) and $^{31}P\{H\}$ NMR (162 MHz, 298 K, $CDCl_3$)

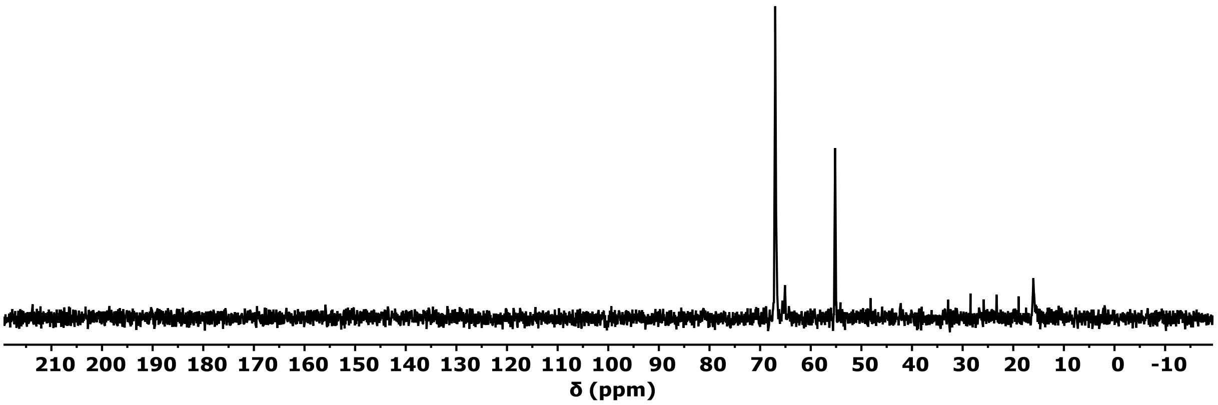

$^{13}C\{H\}$ NMR (101 MHz, 298 K, H_2O/D_2O 9:1)

P7 P(EtPPn-*grad*-EPP)

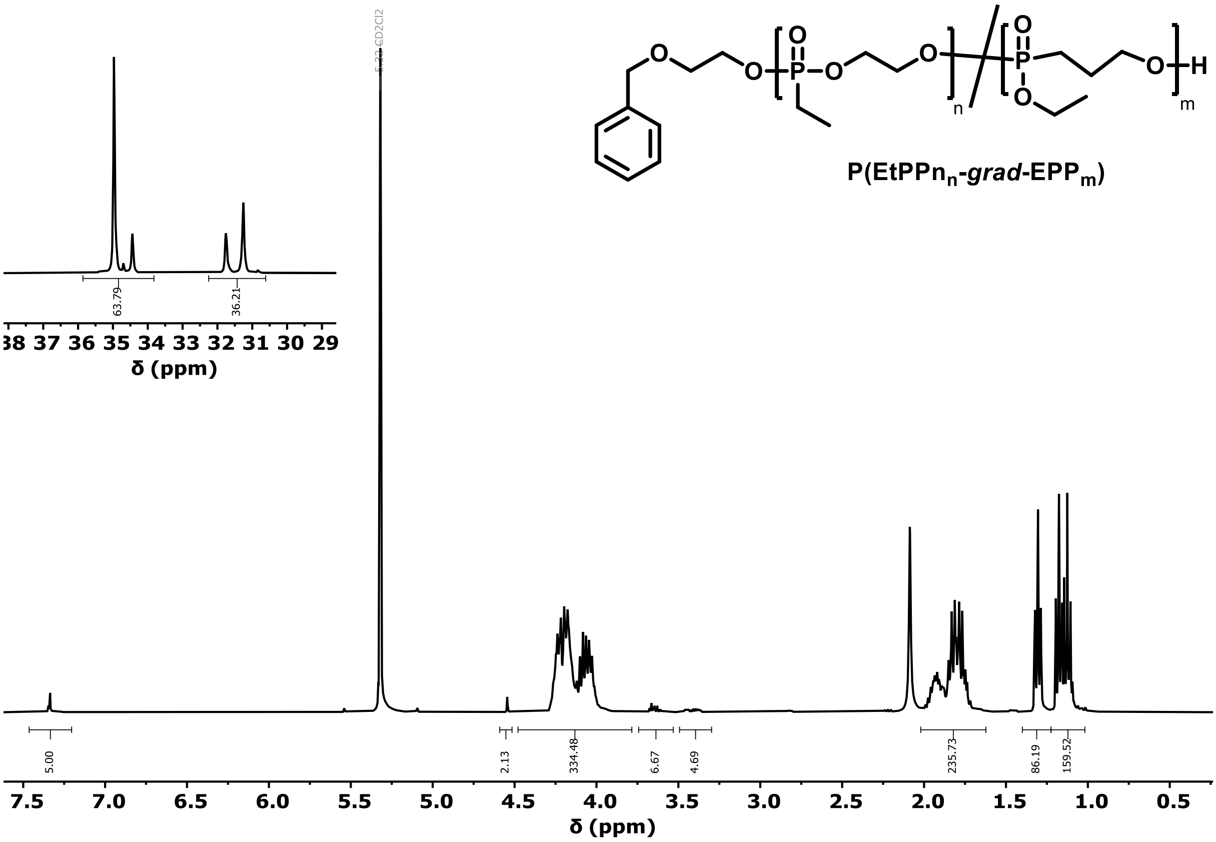

^{1}H NMR (400 MHz, 298 K, CD_2Cl_2) and $^{31}P\{H\}$ NMR (162 MHz, 298 K, CD_2Cl_2)

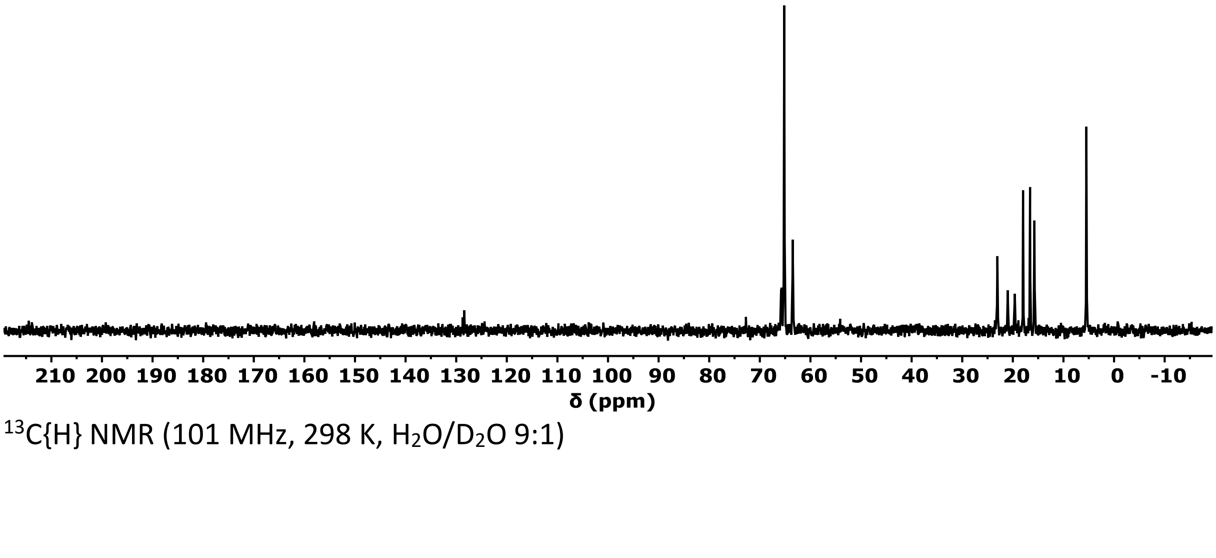

$^{13}C\{H\}$ NMR (101 MHz, 298 K, H_2O/D_2O 9:1)

P8 P(ETP-*grad*-EPP)

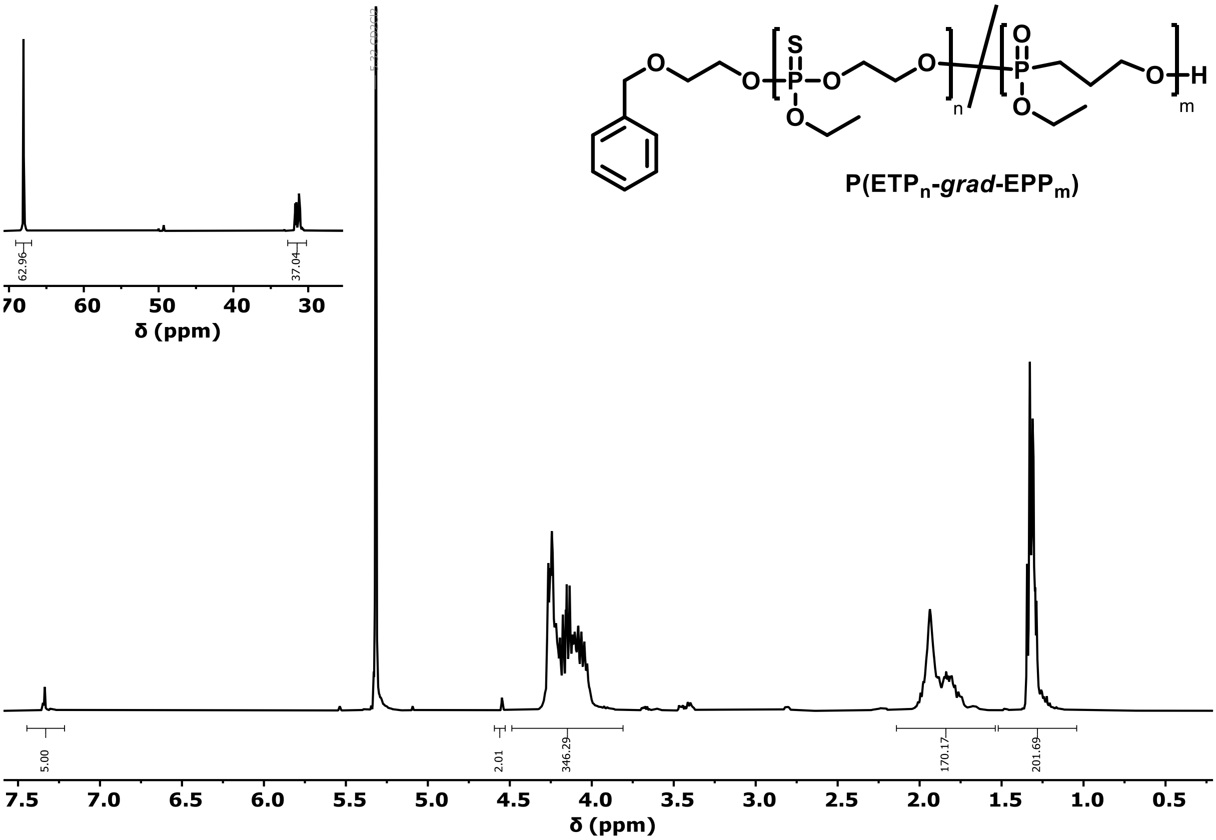

^{1}H NMR (400 MHz, 298 K, CD_2Cl_2) and $^{31}P\{H\}$ NMR (162 MHz, 298 K, CD_2Cl_2)

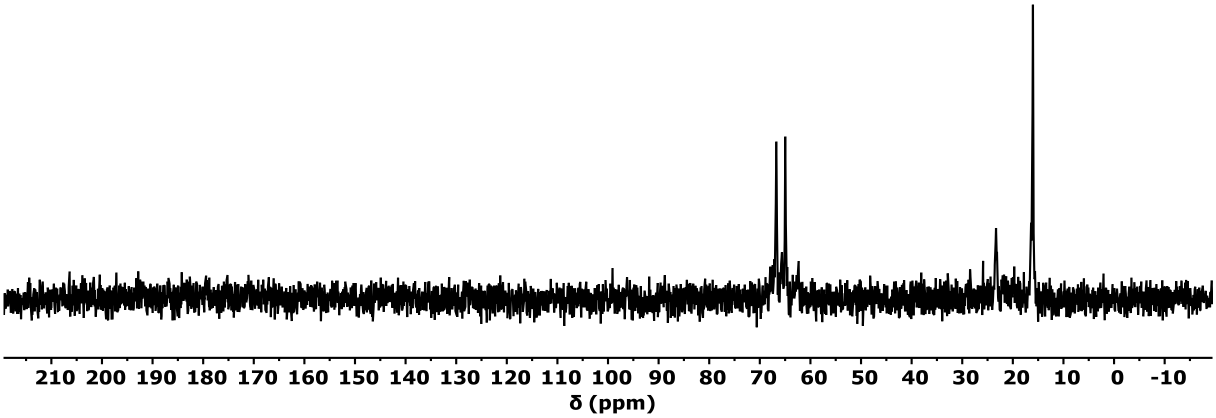

$^{13}C\{H\}$ NMR (101 MHz, 298 K, H_2O/D_2O 9:1)

P9 P(EtPPn-*grad*-ETP-*grad*-EPP)

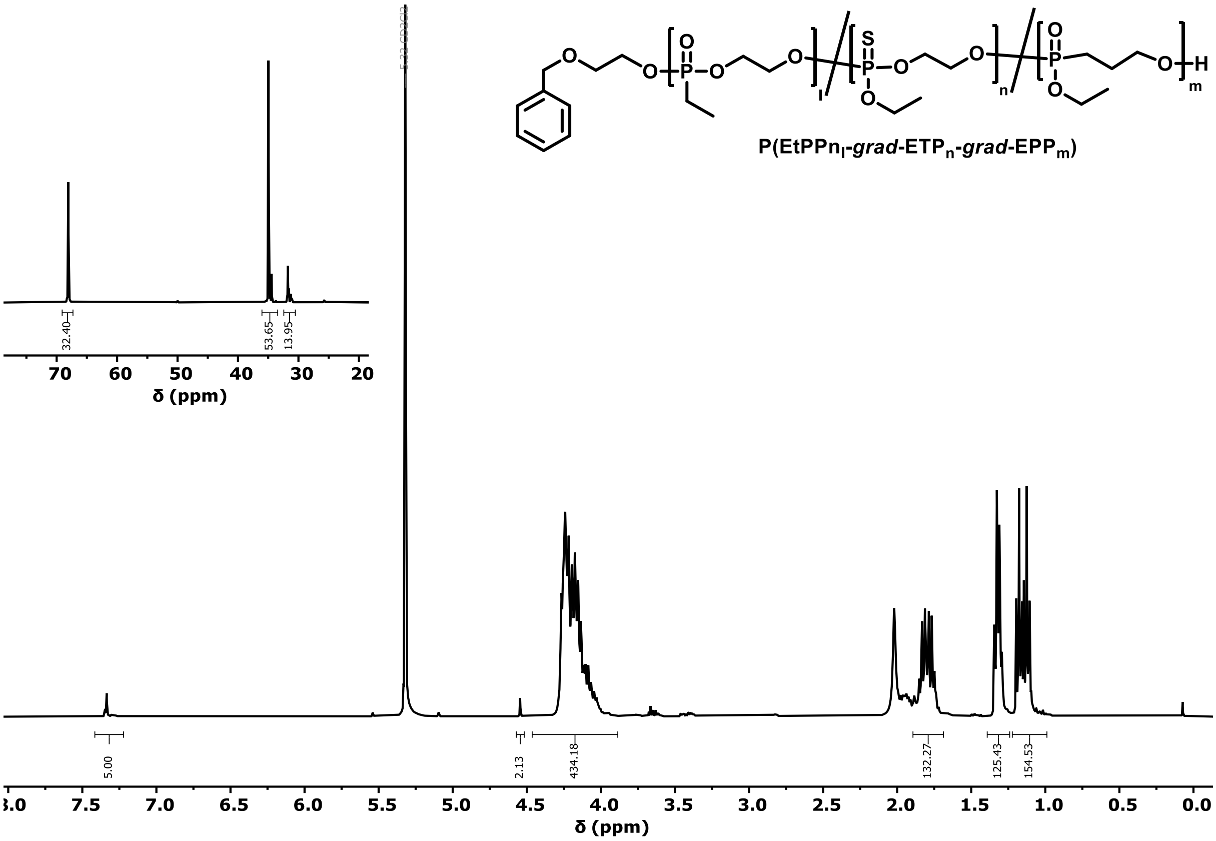

^{1}H NMR (400 MHz, 298 K, CD_2Cl_2) and ^{31}P{H} NMR (162 MHz, 298 K, CD_2Cl_2)

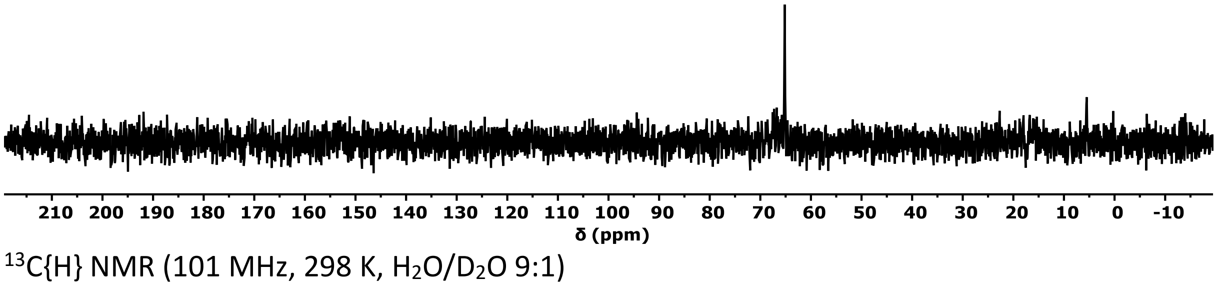

^{13}C{H} NMR (101 MHz, 298 K, H_2O/D_2O 9:1)

P10 P(EtPPn-*grad*-EPP-*grad*-ETP)

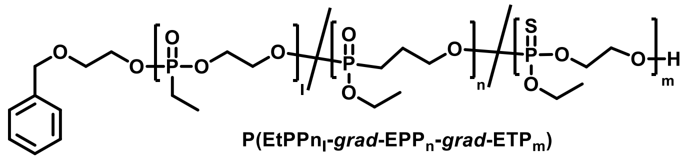

P(EtPPn$_l$-*grad*-EPP$_n$-*grad*-ETP$_m$)

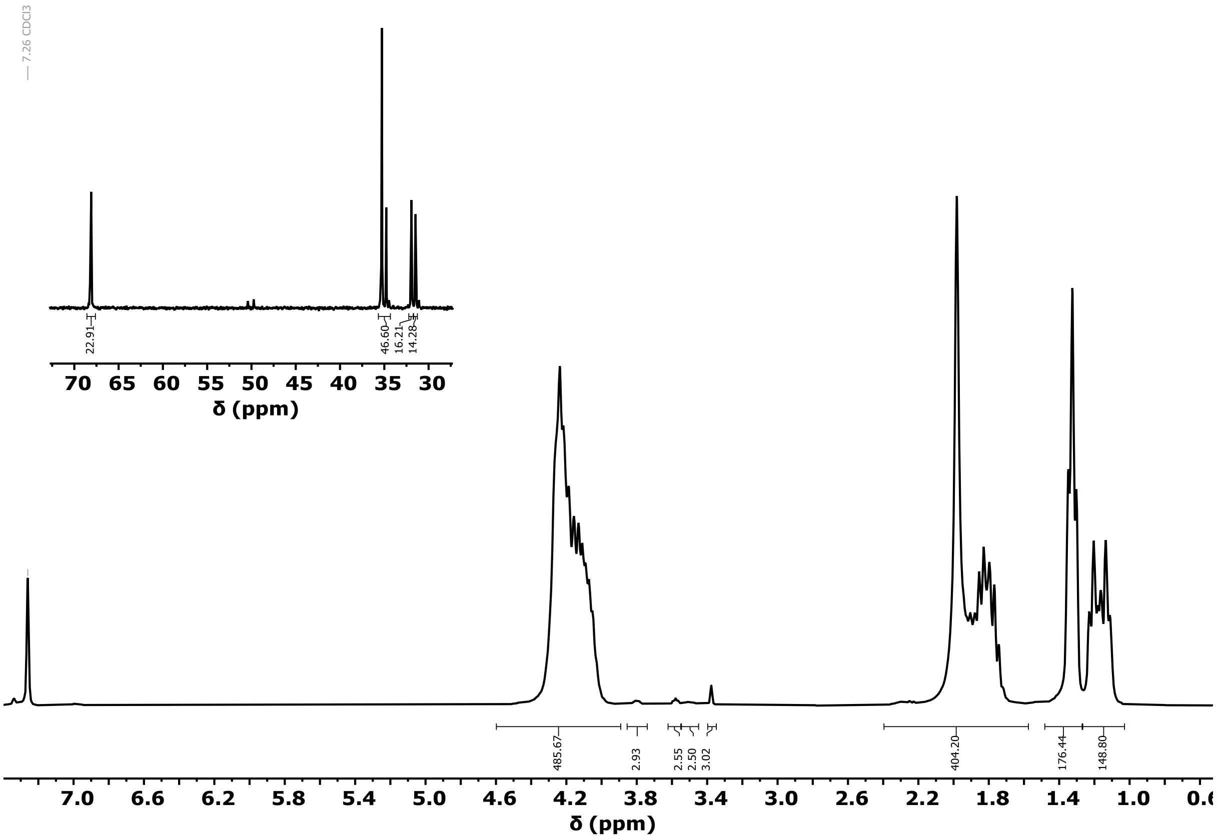

^{1}H NMR (400 MHz, 298 K, $CDCl_3$) and ^{31}P{H} NMR (162 MHz, 298 K, $CDCl_3$)

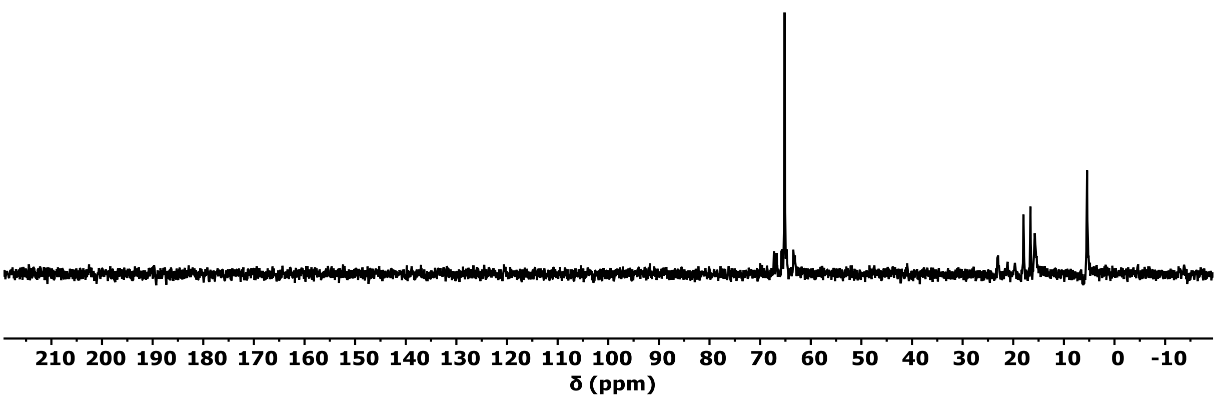

^{13}C{H} NMR (101 MHz, 298 K, H_2O/D_2O 9:1)
